# Interfering small ubiquitin modifiers (SUMO) improves the thermotolerance of apple by facilitating the activity of MdDREB2A

**DOI:** 10.1007/s44154-023-00089-y

**Published:** 2023-05-11

**Authors:** Zeyuan Liu, Ningning Bian, Jianyan Guo, Shuang Zhao, Abid Khan, Baohua Chu, Ziqing Ma, Chundong Niu, Fengwang Ma, Ming Ma, Qingmei Guan, Xuewei Li

**Affiliations:** 1 State Key Laboratory of Crop Stress Biology for Arid Areas/Shaanxi Key Laboratory of Apple, College of Horticulture, Northwest A&F University, Yangling, 712100 Shaanxi China; 2 Department of Horticulture, The University of Haripur, Haripur, 22620 Pakistan; 3 Gansu Academy of Agricultural Sciences, Lanzhou, 730000 Gansu China

**Keywords:** Apple, SUMO, Thermotolerance, MdDREB2A, Gene expression

## Abstract

Heat stress, which is caused by global warming, threatens crops yield and quality across the world. As a kind of post-translation modification, SUMOylation involves in plants heat stress response with a rapid and wide pattern. Here, we identified small ubiquitin modifiers (SUMO), which affect drought tolerance in apple, also participated in thermotolerance. Six isoforms of SUMOs located on six chromosomes in apple genome, and all the SUMOs were up-regulated in response to heat stress condition. The *MdSUMO2* RNAi transgenic apple plants exhibited higher survival rate, lower ion leakage, higher catalase (CAT) activity, and Malondialdehyde (MDA) content under heat stress. MdDREB2A, the substrate of MdSUMO2 in apple, was accumulated in *MdSUMO2* RNAi transgenic plants than the wild type GL-3 at the protein level in response to heat stress treatment. Further, the inhibited SUMOylation level of MdDREB2A in *MdSUMO2* RNAi plants might repress its ubiquitination, too. The accumulated MdDREB2A in *MdSUMO2* RNAi plants further induced heat-responsive genes expression to strengthen plants thermotolerance, including *MdHSFA3*, *MdHSP26.5*, *MdHSP18.2*, *MdHSP70*,* MdCYP18-1* and *MdTLP1*. In summary, these findings illustrate that interfering small ubiquitin modifiers (SUMO) in apple improves plants thermotolerance, partly by facilitating the stability and activity of MdDREB2A.

## Introduction

Global climate change is a big threat for the balance of terrestrial ecosystem, including the continuously increased temperature of ocean and continent, unpredictable rainfall or drought patterns, and extreme weather (Li et al., 2022a; Niu et al. 2022; Smith and Gregory 2013; Wheeler and von Braun 2013). Among the aforementioned climate changes, greenhouse gas-induced global warming poses a significant threat to agriculture production (Geng et al. 2020; Walters et al. 2022; White et al. 2006). Previous studies found that heat stress contributes to overall yield losses of about 40% in wheat (White et al. 2006), about 1–1.7% loss in maize for every raise in temperature above 30 °C (Lobell et al. 2011a), and a 3–7% loss of corn and soybeans with every degree of increased heat. From 1980 to 2008, the reduction in the yield of maize and wheat were 3.8% and 5.5%, respectively, due to global warming (Huang et al. 2019; Lobell et al. 2011b). Additionally, higher temperature and drought events usually coincide more often due to nowadays climate change, that cause the plants more vulnerable to insects and disease infections and finally magnify the yield loss (Dai 2013; Kumar et al. 2019). According to the records of NOAA (National Oceanic and Atmospheric Administration, www.noaa.gov/climate), earth’s temperature has risen by 0.08 °C per decade since 1880 and the last nine years from 2013 to 2021 ranked among the warmest years. While, July 2022 was among the top 10 warmest Julys on record for several continents, and almost the whole world is undergoing the heat-waves for now. So, the discovery of the thermotolerant genes for laying the breeding foundation of heat-tolerant cultivars is urgent need of current research.

Apple is a perennial temperate woody plant, which can survive through high temperature (Huo et al. 2021, 2020). Despite this, the extreme high temperatures cause a significant loss of economic benefit for apple orchards, including the loss of fruit quality and quantity. Direct sunlight can easily destroy the apple fruits due to high temperature, that is known as sunburn with tissue discoloration, yellowing, browning, and necrosis (Torres et al. 2013). Heat stress also affects the quality parameters of apple fruits, including fruit shape, texture, water content, and sugar concentration (Franco et al. 1992; Torres et al. 2017). Apart from the effects on fruit, heat stress can also lead to the unavoidably biochemical and physiological changes in apple trees, including accumulation of ROS (Reactive Oxygen Species) and MDA (malonaldehyde), activated antioxidant enzyme activities, elevated leaf electrolyte leakage, changed stomatal movement, decreased photosynthesis capacity, phytohormones homeostasis, anthocyanin metabolism and enhanced autophagic activity (Dong et al. 2021; Fang et al. 2019; Huo et al. 2021, 2020; Torres et al. 2017). Additionally, the roles of various genes associated with the apple heat stress response have been revealed; and the HSFs (heat shock transcription factors) are the most investigated of these genes. In apple genome, 25 HSFs have been identified and proved to participate in heat stress response (Giorno et al. 2012). Especially, MdHSF3b and MdHSF4a, two apple positive regulators under high temperature condition, bind to the heat shock cis-element of *MdCOL4* promoter. Then, the induced *MdCOL4* enhances its inhibitory effect to regulate the expression of genes involved in anthocyanin biosynthesis under high temperature conditions (Fang et al. 2019). MdNup62, an interacted protein of MdHSFs, negatively regulates the heat stress by inhibiting the expression of HSPs (Zhang et al. 2022). Moreover, the VQ motif-containing protein-coding gene (*MdVQ37*) is a negative regulator of apple basal thermotolerance by affecting salicylic acid homeostasis and heat related genes expression (Dong et al. 2021). Furthermore, apple basal thermotolerance can also be affected by autophagy, and overexpression of *MdATG18a* enhanced the apple thermotolerance by decreasing chloroplasts damage (Huo et al. 2020). Since the vital roles of autophagy played in apple heat stress response, MdATG8i also has been proved to be a positive regulator under high temperature environment, and the same role of the MdATG8i-interacting protein, MdHARBI1 (Huo et al. 2021). However, research on apple heat stress response is still limited, and more research is needed on the gene functions involved in apple thermotolerance.

SUMO, a small protein of approximately 100–115 amino acids, usually attached to a protein for participating in plants biotic and abiotic stress response (Castro et al. 2012; Chang and Yeh 2020; Hay 2005). The attachment of SUMO to the substrates is known as a kind of protein post-translation modification—SUMOylation (Morrell and Sadanandom 2019; Tempe et al. 2008; Wang et al. 2020). SUMOylation is a rapid and efficient manner for plants biotic and abiotic stress response by affecting the functions of target proteins, including stability, transcription activity, subcellular trafficking, genome integrity, and chromatin-remodeling (Castro et al. 2012; Guerra et al. 2015; Miller et al. 2010; Miller and Vierstra 2011). Previous studies have found various potential target substrates of SUMO under heat stress condition, like HSPs, HSFs, DREB2A, WRKYs, TPL, SUVH9, and histone H2B (Castro et al. 2012; Guerra et al. 2015; Miller et al. 2010; Miller and Vierstra 2011; Rytz et al. 2018). However, the impact of SUMOylation of these target proteins is largely unresolved, with the exception of the transcription factors HSFA2 and DREB2A. Arabidopsis HSFA2 is a positive regulator of plants acquired thermotolerance, which is SUMOylated at K315. The transcriptional activation of HSFA2 on the down-stream HSPs promoters is repressed by overexpression of AtSUMO1 (Cohen-Peer et al. 2010). DREB2A is another positive regulator of heat stress, and its SUMOylation enhances the protein stability to confer plants acquired thermotolerance (Wang et al. 2020). Additionally, the DREB2A is also known as a target substrate of SUMOs in apple and its SUMOylation responds to drought stress condition via a fine-tunning manner (Li et al., 2022a), that is in contrast with Arabidopsis. However, how SUMOs participate in apple heat stress has not been reported yet.

In this study, we found that interfering SUMO expression could improve thermotolerance of transgenic apple plants. The *MdSUMO2* RNAi plants showed enhanced capability of scavenging H_2_O_2_ and maintaining integrity of plasma membranes. Furthermore, we found that the SUMO target substrate, MdDREB2A was accumulated more in the *MdSUMO2* RNAi plants compared to the wild type GL-3 under heat stress condition. Inhibited SUMOylation of MdDREB2A also suppressed its ubiquitination and then facilitated the protein stability of MdDREB2A. Since the positive role of MdDREB2A in plants heat stress response, we concluded that the accumulated MdDREB2A partly contributed to the thermotolerance of *MdSUMO2* RNAi plants. Moreover, the elevated expression of heat-responsive genes acted as the down-stream of MdDREB2A might be another reason for the improved thermotolerance of *MdSUMO2* RNAi plants. Taken together, we have characterized the improved thermotolerance of *MdSUMO2* RNAi plants by enhancing the protein stability of MdDREB2A and activating the down-stream heat-responsive genes expression.

## Results

### Apple small ubiquitin modifiers are involved in heat stress

**Fig. 1:**
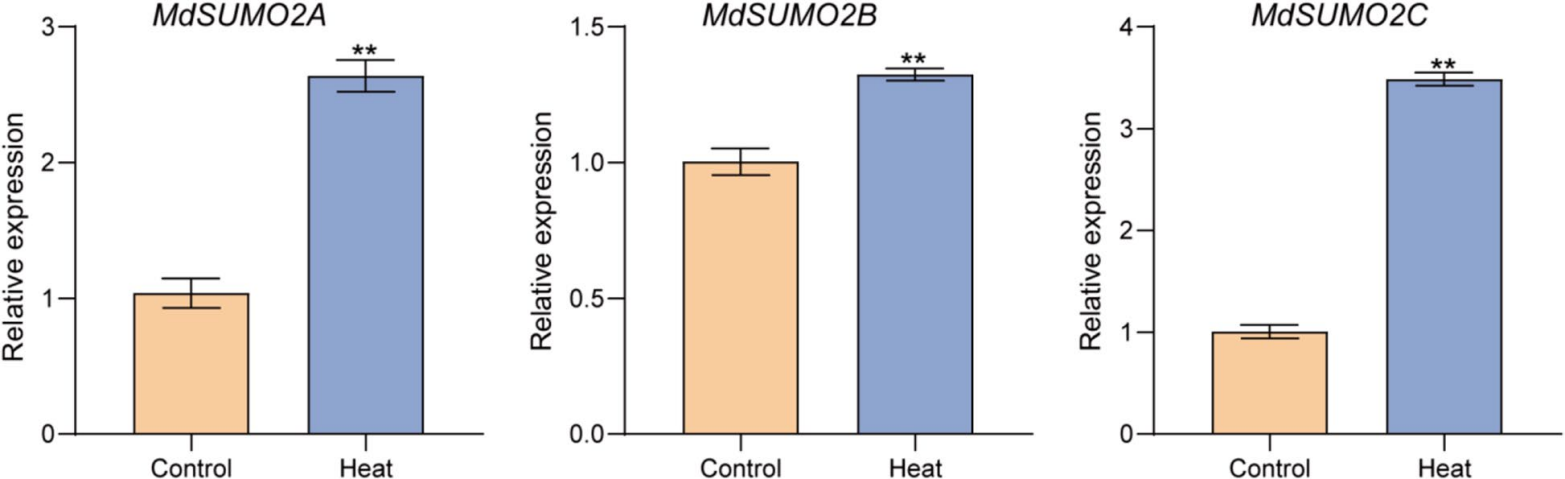
Expression patterns of *MdSUMO2*s in apple under heat stress. Error bars indicate SD, *n* = 3. Asterisks indicate statistically significant differences. Student’s t test was performed and statistically significant differences were indicated by *P ≤ 0.05, **P ≤ 0.01

Previous study found that there were six small ubiquitin modifiers (SUMO) in apple genome, and due to the whole genome duplication, these SUMOs located on six chromosomes shared very similar sequences (Li et al., 2022a). Apple *MdSUMOs* modulate drought stress response with a fine-tuning manner. To further explore whether *MdSUMOs* function under heat stress in apple, firstly, GL-3 apple plants were subjected to heat exposure (45 °C) for 2 h. Due to the almost identical coding sequences, we separated apple SUMOs to MdSUMO2A (on chromosome 3 and 11), MdSUMO2B (on chromosome 7 and 17), and MdSUMO2C (on chromosome 5 and 10). The qRT-PCR results showed that three *MdSUMO2*s were up-regulated with varying degrees under heat stress condition (Fig. 1), implying the similar roles of *MdSUMO2* involved in heat stress.

### Interfering small ubiquitin modifiers (SUMO) improves thermotolerance of apple

**Fig. 2:**
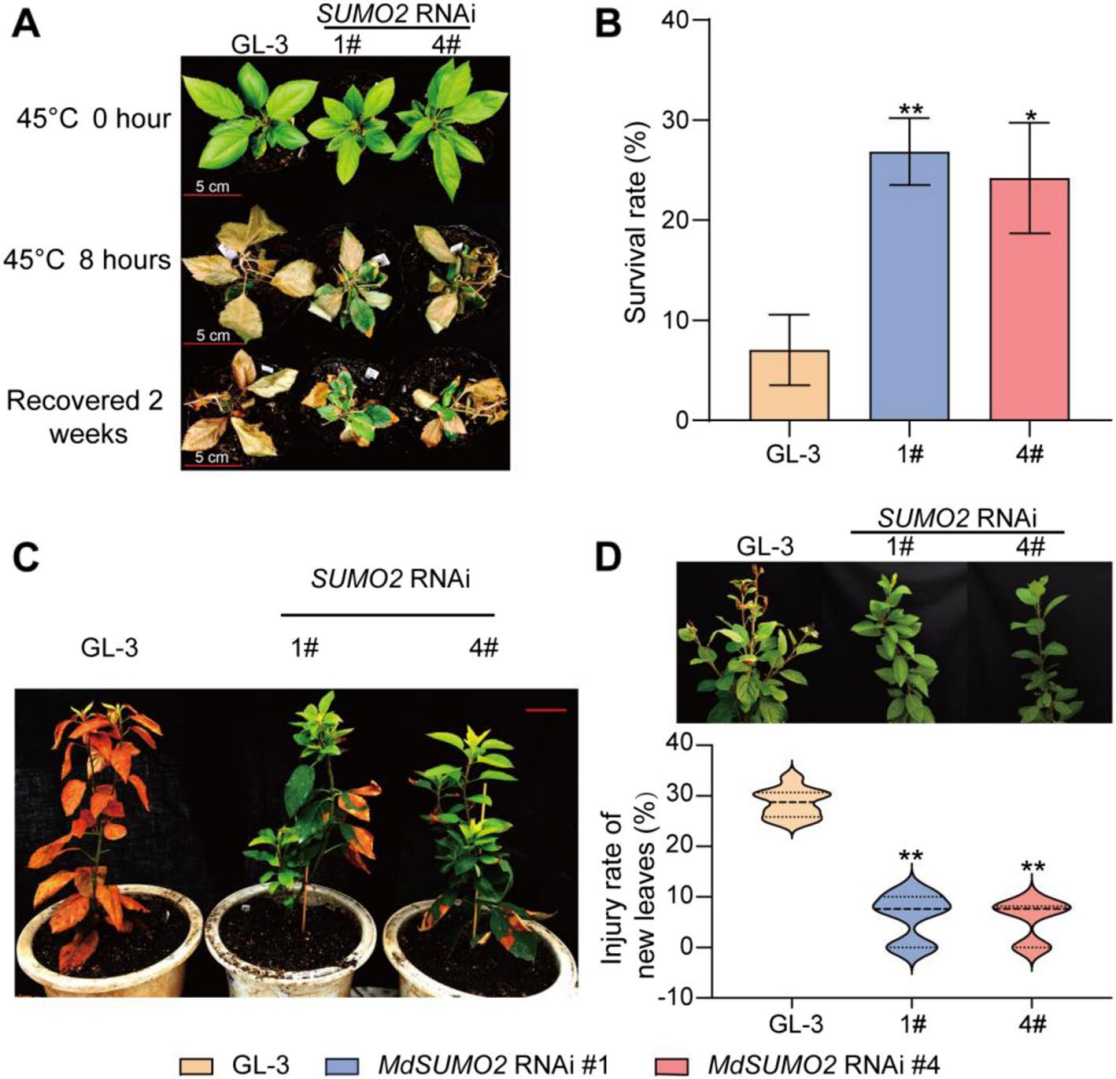
The *MdSUMO2* RNAi transgenic apple plants are more tolerance than the wild type (GL-3) under heat stress condition. **A**, Morphology of one-month-old *MdSUMO2* RNAi transgenic apple plants and GL-3 under heat stress for 8 h. **B**, The survival rate in A. **C**, Morphology of 3-month-old *MdSUMO2* RNAi transgenic apple plants and GL-3 under 40 °C for 7 days. **D**, The new leaves of GL-3 and transgenic plants under heat stress (up, the morphology), and the statistical data (down, the injury rate of new leaves). Error bars indicate SD, *n* = 24 in A and B, and *n* = 10 in D. Asterisks indicate significant differences between *MdSUMO2* RNAi transgenic apple plants and GL-3 plants in each group (control and heat treatment). Student’s t test was performed and statistically significant differences were indicated by *P ≤ 0.05, **P ≤ 0.01

We generated apple transgenic plants of *MdSUMO2* RNAi, which knocked down the expression of all *SUMO*s mentioned in the previous study (Li et al., 2022a). Initially, one-month-old GL-3 and *MdSUMO2* RNAi apple seedlings were used as experimental materials. When exposed to 45 °C for 8 h, almost all the leaves of the wild type—GL-3 plants wilted and turned brown. In contrast, scorching of the *MdSUMO2* RNAi leaves partially occurred with symptoms like edges dry out and turn brown (Fig. 2A). Two weeks later, more than 20% *MdSUMO2* RNAi transgenic seedlings recovered with new leaves, however, only 5% of the wild GL-3 survived and recovered (Fig. 2B). Similar phenotypes were also found in three-month-old GL-3 and *MdSUMO2* RNAi transgenic apple seedlings exposed to the outdoor high temperature environment for 7 days. At 40 °C, the leaves of the GL-3 plants mostly displayed the symptoms of dehydration and shriveled, nevertheless, the leaves of the *MdSUMO2* RNAi transgenic plants remained green and vigorous (Fig. 2C). Additionally, eight-month-old GL-3 and *MdSUMO2* RNAi plants raised in a greenhouse suffered from high temperature at noon when the temperature reached to 50 °C within a short time. A short exposure of heat resulted the serious injury of new leaves of GL-3 (about 28.6%), however few new leaves of *MdSUMO2* RNAi plants exhibited yellowing and dehydration (Fig. 2D).

### Knock-down of the MdSUMO2s in apple enhanced the stability of plasma membranes

**Fig. 3:**
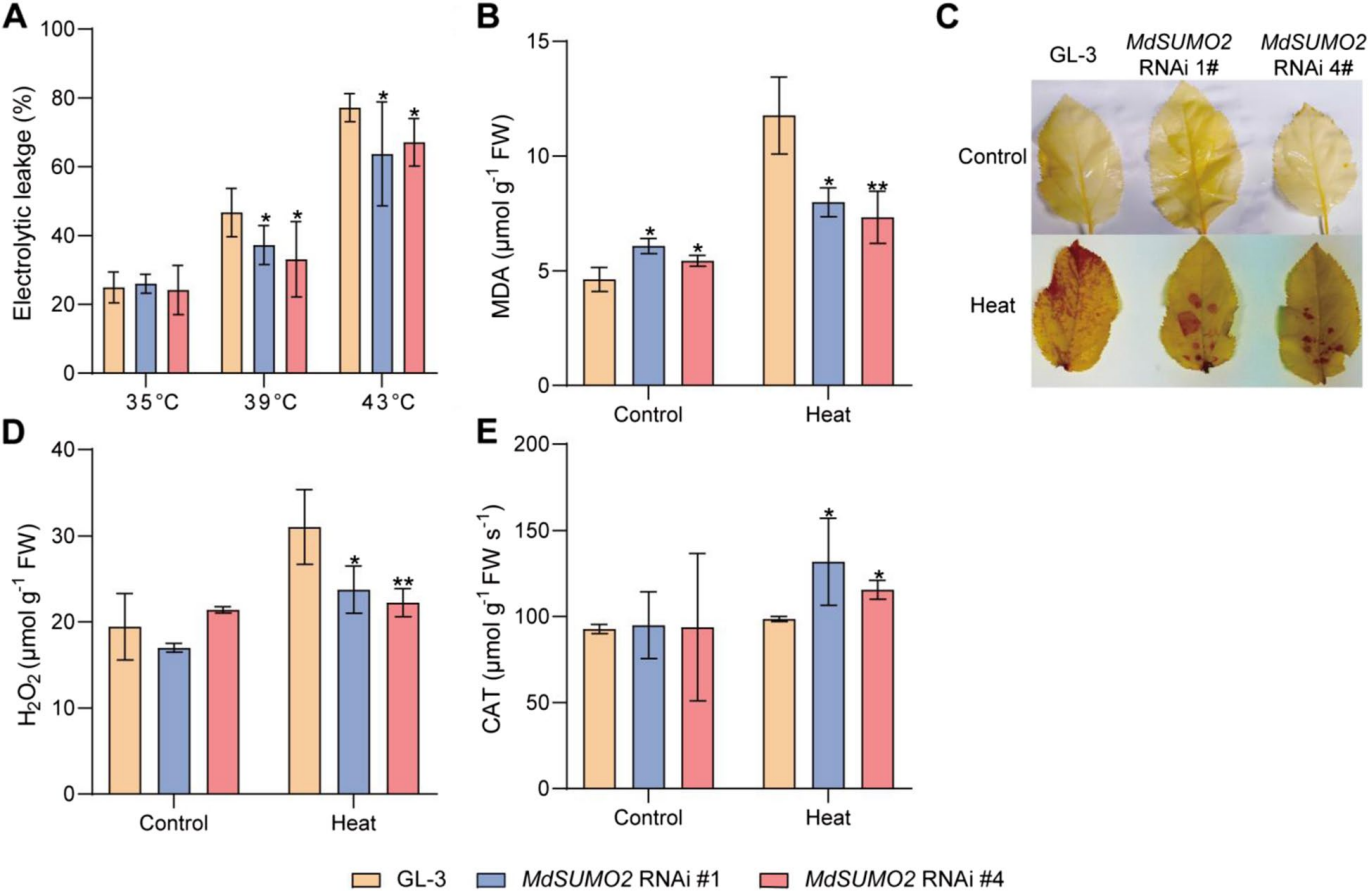
The activated catalase contributes the thermotolerance of *MdSUMO2* RNAi transgenic apple plants than GL-3. **A**, The electrolytic leakage of leaves under high temperature stress. **B**, Malondialdehyde (MDA) concentration in GL-3 and transgenic apples treated with or without high temperature. **C**, DAB staining of *MdSUMO2* RNAi transgenic apple plants and GL-3 under control and heat treatment. **D**, H_2_O_2_ contents of GL-3 and transgenic plants under control and heat treatment. E, Catalase (CAT) activity in *MdSUMO2* RNAi transgenic apple and GL-3 under control and heat treatment. Error bars indicate SD, *n* = 3. Asterisks indicate significant differences between *MdSUMO2* RNAi transgenic apple plants and GL-3 plants in each group (control and heat treatment). Student’s t test was performed and statistically significant differences were indicated by *P ≤ 0.05, **P ≤ 0.01

Previously, it has been reported that cell plasma membranes are considered as heat stress sensors (Török et al. 2014), so we further investigated the stability of plasma membranes of GL-3 and *MdSUMO2* RNAi plants under heat stress condition. As Fig. 3A showed, apple plants could suffer high temperature at 35 °C. While the leaves of both GL-3 and *MdSUMO2* RNAi plants started ion leakage at 39 °C. The electrolytic leakage aggravated when the temperature reached to 43 °C. During the heat stress condition, the electrolytic leakage of *MdSUMO2* RNAi plants was much lower than the GL-3, indicating the relative stability of plasma membranes of *MdSUMO2* RNAi leaves (Fig. 3A). The malondialdehyde (MDA) content acts as a marker of lipid peroxidation (Del Rio et al. 2005). We found that high temperature treatment enhanced the MDA concentration of the GL-3 and *MdSUMO2* RNAi plants, while the MDA content of the *MdSUMO2* RNAi plants was lower than GL-3 (Fig. 3B). This result showed the mild damage to *MdSUMO2* RNAi plants caused by high temperature than GL-3 plants. Abiotic stress usually leads to ROS burst in plants (Niu et al. 2022). Our results of DAB staining showed that heat stress caused the accumulation of H_2_O_2_ content in apple plants. As compared to the GL-3 plants, the leaves of the *MdSUMO2* RNAi plants were lightly stained with brown coloration (Fig. 3C). Meanwhile, we determine the H_2_O_2_ contents of GL-3 and transgenic plants under control and heat treatment. The results were in agreement with the DAB staining (Fig. 3D). However, in case of NBT staining, no significant differences were observed between the GL-3 and *MdSUMO2* RNAi plants (data not shown). Furthermore, we detected the activity of catalase (CAT), which is responsible for H_2_O_2_ scavenging. The results showed that the CAT activity was significantly increased in *MdSUMO2* RNAi plants than GL-3 after heat stress (Fig. 3E). Altogether, these findings indicated that knock-down of the *MdSUMO2*s in apple enhanced the thermotolerance by reducing the accumulation of H_2_O_2_.

### Heat caused the accumulation of MdDREB2A in *MdSUMO2* RNAi transgenic apple plants

**Fig. 4:**
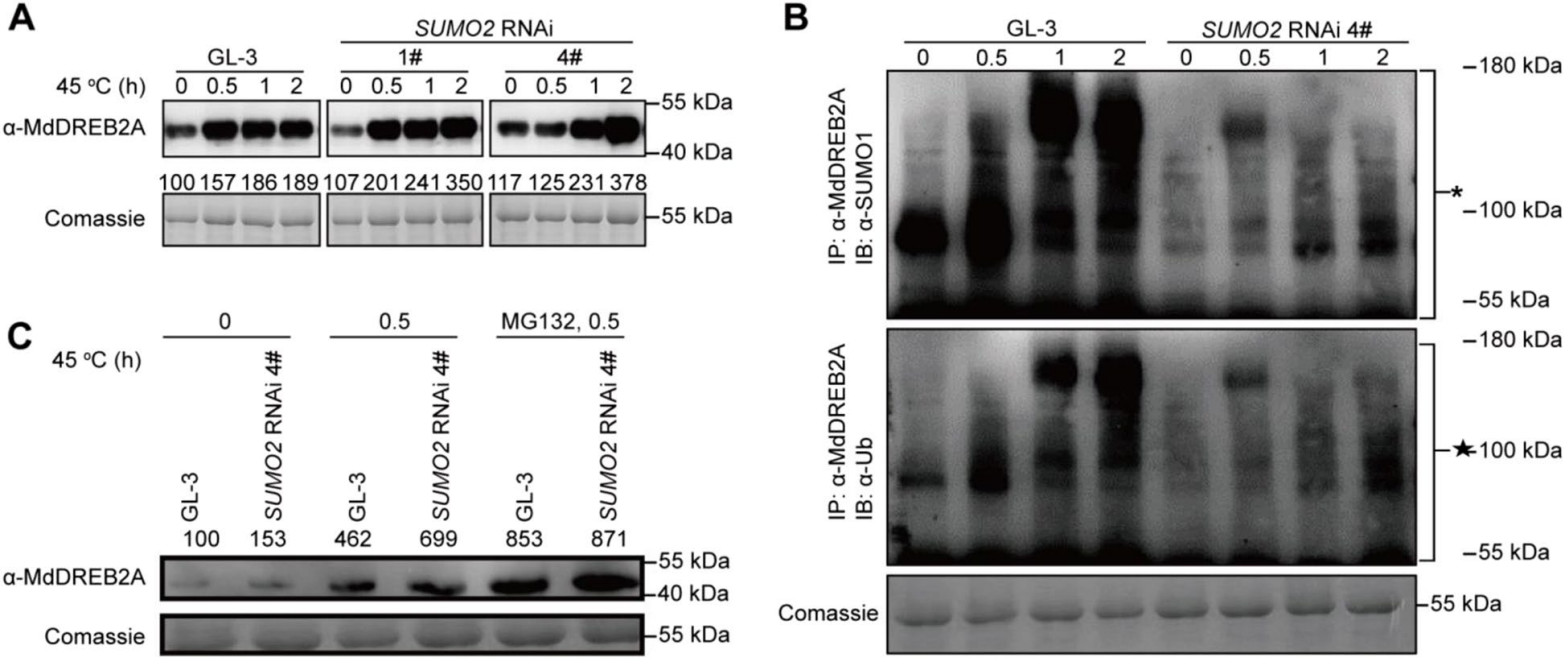
The transcription factor MdDREB2A was accumulated in *MdSUMO2* RNAi transgenic apple plants than GL-3. A, The proteins of MdDREB2A in *MdSUMO2* RNAi transgenic plants were induced more than GL-3 under heat stress. B, SUMOylation and ubiquitination of MdDREB2A in GL-3 and *MdSUMO2* RNAi transgenic plants in response to heat stress treatment. * indicates SUMOylated substrates; ★indicates ubiquitinated substrates. C, The stability of MdDREB2A was affected by 26S proteasome mediated degradation in response to heat stress condition. MG132 was applied as the inhibitor of 26S proteasome mediated ubiquitination

Under heat stress condition, the transcription factor DREB2A is regarded as a positive regulator (Sakuma et al. 2006). SUMOylation of DREB2A facilitates its protein stability and improve Arabidopsis thermotolerance (Wang et al. 2020). Nevertheless, SUMOylation of apple MdDREB2A mediates its ubiquitination and following degradation under drought stress condition (Li et al., 2022a). Here, when exposed to heat stress, we found that apple MdDREB2A was quickly activated, and that *MdSUMO2* RNAi transgenic apple plants accumulated more of it than GL-3 (Fig. 4A). As SUMOylation can affect the protein stability, so we examined the SUMOylation level of MdDREB2A protein in both GL-3 and *MdSUMO2* RNAi transgenic plants under control and heat stress conditions. The Fig. 4B shows that 45 °C heat stress treatment significantly increased the level of SUMO conjugates to MdDREB2A in GL-3 plants, which was much lower than in *MdSUMO2* RNAi transgenic plants. Because of the tight crosstalk between SUMOylation and ubiquitination, we further investigated the ubiquitination level of MdDREB2A in response to heat stress condition. The results showed a synergistic effect of SUMOylation and ubiquitination on MdDREB2A, as the ubiquitination level of MdDREB2A increased lower in *MdSUMO2* RNAi transgenic plants than GL-3 in response to heat stress (Fig. 4B). To investigate the mechanism of SUMO-mediated DREB protein degradation further, we treated plants with MG132, a 26S proteasome pathway inhibitor. The western bolt result demonstrated that MdDREB2A accumulated more in *MdSUMO2* RNAi transgenic apple plants than the wild type GL-3 under heat stress treatment significantly. MG132 was applied to check the stability of MdDREB2A was whether associated with ubiquitination, and the result showed that the MdDREB2A protein degradation was inhibited both in GL-3 and *MdSUMO2* RNAi transgenic lines. However, MdDREB2A still accumulated more than GL-3 (Fig. 4C). The analysis of the stability of the MdDREB2A protein under heat stress revealed that the 26S proteasome pathway was involved in the SUMOylation-mediated degradation of MdDREB2A.

### Silencing of *MdSUMO2* in apple upregulated the down-stream genes of MdDREB2A under heat stress

**Fig. 5:**
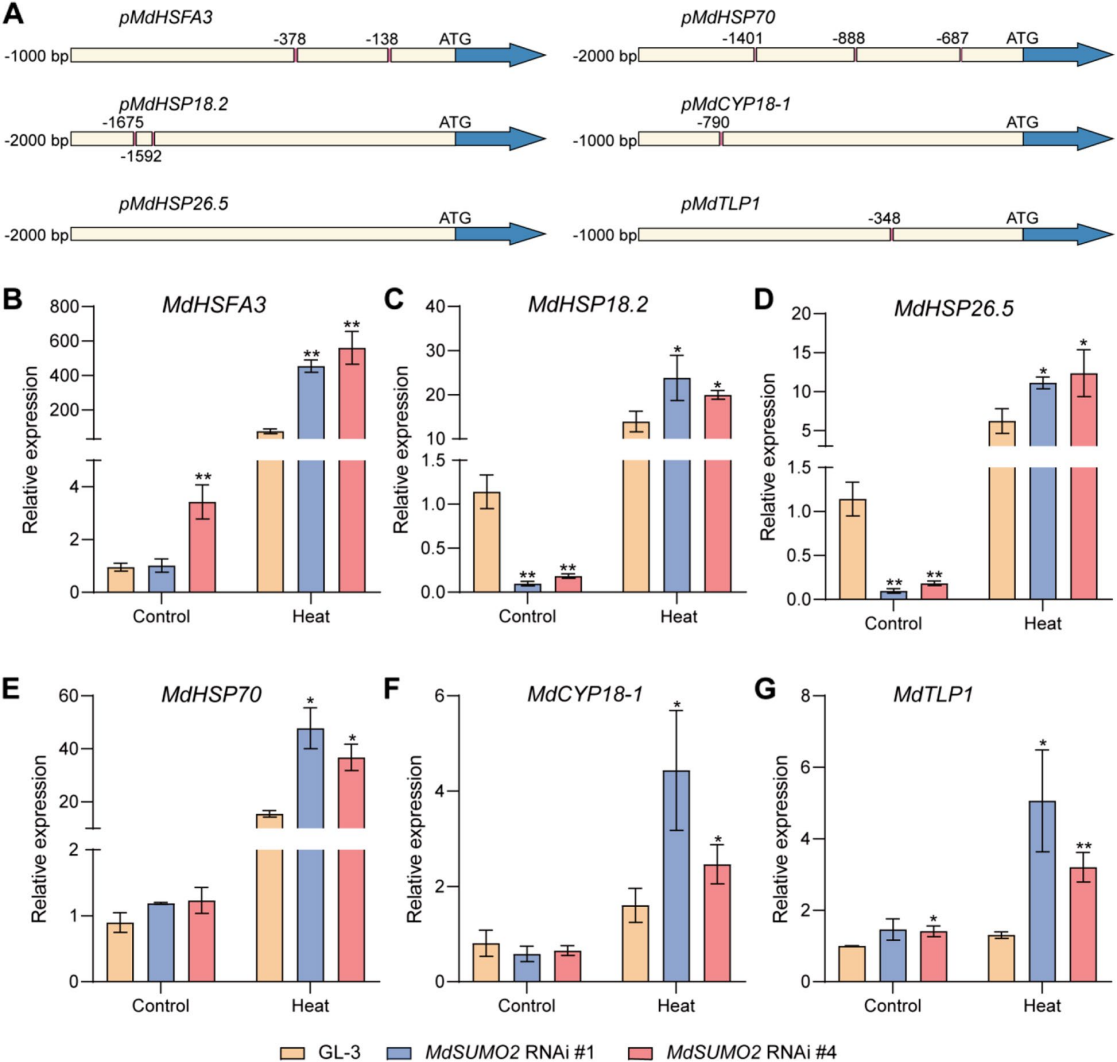
The transcription factor MdDREB2A activates the expressions of down-stream genes related to heat response. **A**, A scheme of the down-stream genes. The coding region is shown in indigo and beige indicates the promoter and 5 ‘UTR. The binding sites of MdDREB2A are shown with pink. **B**-**G**, Expression of MdDREB2A downstream genes in *MdSUMO2* RNAi transgenic apple plants and GL-3 plants under control and heat treatment. Error bars indicate SD, *n* = 3. Asterisks indicate significant differences between *MdSUMO2* RNAi transgenic apple plants and GL-3 plants in each group (control and heat treatment). Student’s t test was performed and statistically significant differences were indicated by *P ≤ 0.05, **P ≤ 0.01

We next explored whether MdDREB2A accumulation affected the down-stream genes expression in response to heat stress. Six homologous genes of Arabidopsis which were proved to be down-stream of DREB2A were selected to detect mRNA expression levels (Sakuma et al. 2006). Except *MdHSP26.5*, other five genes were found the conserved DRE motif on their promoters (Fig. 5A). There is only one DRE motif on the promoters of *MdTLP1* and *MdCYP18.1*, two motifs on *MdHSP18.2* and *MdHSFA3*, and three motifs on *MdHSP70*. We further used quantitative RT-PCR to confirm the expression patterns of these heat stress responsive genes. As Fig. 5B showed, *MdHSFA*, *MdHSP70*,* MdCYP18-1* and *MdTLP1* were found the similar expression level under control condition, but induced significantly more in *MdSUMO2* RNAi transgenic plants than GL-3. The expression levels of *MdHSP18.2* and *MdHSP26.5* were lower in *MdSUMO2* RNAi transgenic plants under control condition, but they were induced clearly higher in *MdSUMO2* RNAi transgenic plants in response to heat stress condition (Fig. 5B). These results suggest that MdSUMOs are involved in heat stress response by inducing heat-responsive genes expression which acted as the down-stream genes of MdDREB2A.

## Discussion

Global warming is making heat waves hotter, longer, and more common. Plants have evolved multiple characteristics to adapt the heat stress condition since they are sessile organisms (Walters et al. 2022; Zhu 2016). At the organellar level, leaf properties such as leaf color and leaf angle are changed when plants exposure to heat stress, following with the stomatal closure, inhibited photosynthesis and carbon assimilation (Blum 1986; Slattery and Ort 2019). Heat stress during flowering, gametogenesis and loral meristem stages especially results in a huge loss of crop yield and quality (Jagadish et al. 2021; Torres et al. 2013). Severe heat stress can even lead to the death of plant. At the molecular level, the heat stress caused the damage of cellular phospholipid membranes can be sensed by a series of integral membrane proteins rapidly (Scharf et al. 2012). Following, the calcium signaling, MAPK activation, ROS, NO, phospholipid signals, protein SUMOylation, and proteasomal degradation initiate physiological and biochemical changes (Hasanuzzaman et al. 2013; Zhu 2016). In our study, we identified that all the SUMOs in apple genome were up-regulated under heat stress condition (Fig. 1), implying the potential role of SUMO in response to heat stress. Furthermore, we found that the *MdSUMO2* RNAi transgenic apple plants exhibited enhanced thermotolerance than the wild type—GL-3 (Fig. 2A-B). The one-month-old *MdSUMO2* RNAi transgenic apple plants showed high survival rate under heat stress condition, and the 3-month-old plants sustained green leaves as compared to the scorched leaves of GL-3 after exposing to heat condition (Fig. 2C). Additionally, the 8-month-old *MdSUMO2* RNAi plants displayed much less injured new shoots than GL-3 in greenhouse condition. Since the cellular phospholipid membranes usually act as an important senser to heat stress, so we investigated the permeability of membranes (Huo et al. 2020; Zhang et al. 2021). The results showed that *MdSUMO2* RNAi transgenic apple plants retained the integrity of the membranes as compared to the GL-3 plants upon heat stress exposure. In a coincide, the MDA content was found much lower in *MdSUMO2* RNAi plants after high temperature treatment. ROS scavenging including H_2_O_2_ and O_2_^−^, are usually regarded as an effective way for plants to adapt heat stress condition (Hasanuzzaman et al. 2013; Naing and Kim 2021). Our result showed lower H_2_O_2_ content in *MdSUMO2* RNAi transgenic apple plants than GL-3, but no significant changes in O_2_^−^ (Fig. 3). Moreover, the catalase activity was found higher in *MdSUMO2* RNAi transgenic apple plants. So, we speculated the activated catalase was responsible for the lower accumulation of H_2_O_2_ content in *MdSUMO2* RNAi plants.

SUMOylation is a kind of post-translation modification, which widely and rapidly participates in plant heat stress response (Castro et al. 2012; Miller and Vierstra 2011; Morrell and Sadanandom 2019). Among various substrates of SUMO, the HSFA2 plays a positive role in heat stress condition. SUMOylation of HSFA2 mediated by SUMO1 exhibits an inactive state in the cell nucleus and deSUMOylation of HSFA2 leads to an active form of this protein when expose to heat stress (Cohen-Peer et al. 2010). SUMOylation also facilitates the importation of chloroplast proteins during heat stress and further regulates the nonphotochemical quenching level of plants (Zheng et al. 2022). Additionally, chromatin-associated SUMOylation regulates the balance between plant development and heat stress responses by controlling the transcriptional switch (Han et al. 2021). Here, we found that MdDREB2A was accumulated more rapidly in *MdSUMO2* RNAi transgenic apple plants as compared to GL-3 under heat stress condition (Fig. 4A). Additionally, DREB2A has been proved to be a positive regulator in plant heat stress response, including Arabidopsis (Sakuma et al. 2006; Wang et al. 2020), maize (Qin et al. 2007), and soybean (Mizoi et al. 2013). Since the DNA-binding capability of DREB2A to the down-stream genes in apple and Arabidopsis are the same in regulating drought stress (Li et al. 2019; Li et al., 2022a), we speculate that the accumulated MdDREB2A in *MdSUMO2* RNAi transgenic apple plants was responsible for their strengthened thermotolerance. Previously it was found that SUMOylation of the MdDREB2A mediated its degradation via the 26S protease pathway through MdRNF4 in response to drought stress condition (Li et al., 2022a). So, we are curious about the role of SUMOylation of MdDREB2A in regulating the stability of MdDREB2A in response to heat stress. We got the similar results for MdDREB2A under drought stress condition, MdDREB2A was both less SUMOylated and ubiquitinated in the *MdSUMO2* RNAi transgenic apple plants than GL-3 under heat stress condition (Fig. 4B). So, we supposed that SUMOylation of MdDREB2A regulated the drought and heat stress response of apple in the same manner, which was different from Arabidopsis under heat condition (Wang et al. 2020). Previous research has suggested that the AtDREB2A protein contains a 30-amino-acid NRD (negative regulatory domain) domain after translation of the AP2 domain. This domain interacts with BPMs (BTB/POZ AND MATH DOMAIN proteins) and DRIP1/2 (DREB2A-INTERACTING PROTEINS 1 and 2) to degrade AtDREB2A (Morimoto et al. 2017; Qin et al. 2008). Under drought or high-temperature stress, the expression of DRIP1/2 and BPMs is reduced, which prevents the hydrolysis of AtDREB2A, thereby promoting its regulation of downstream genes and enhancing plant stress resistance. However, apple MdDREB2A is structurally dissimilar to AtDREB2A except for the AP2 domain, suggesting that there may be inconsistencies in the response of MdDREB2A to stress compared to Arabidopsis. Previously, we discovered that the SUMOylation of apple MdDREB2A could mediate the ubiquitination of MdDREB2A via 26S protease pathway under drought stress condition (Li et al. 2022). In this study, we found that the similar regulation manner of apple MdDREB2A in response to heat stress as drought stress—the accumulation of MdDREB2A in response to heat stress is also controlled by SUMOylation mediated degradation. As a transcription factor, the DREB2A regulates the expression of the down-stream genes by binding to the DRE motif on their promoters (Li et al. 2019; Sakuma et al. 2006). According to the previous study, we identified the homologous genes of Arabidopsis in apple (Sakuma et al. 2006), including *MdHSFA3*, *MdHSP26.5*, *MdHSP18.2*, *MdHSP70*,* MdCYP18-1* and *MdTLP1*. All these genes had DRE motif on their promoters, except *MdHSP26.5*. Furthermore, the qRT-PCR results showed that these genes were induced more in *MdSUMO2* RNAi transgenic apple plants as compared to the GL-3 under heat stress condition, implying their contribution in the thermotolerance of the *MdSUMO2* RNAi plants.

In conclusion, our results demonstrated that interfering small ubiquitin modifiers (SUMO) improves the thermotolerance of apple by facilitating the activity of MdDREB2A. The exposure of *MdSUMO2* RNAi transgenic apple plants to heat stress led to much lighter damage of cellular phospholipid membranes and lower MDA content than GL-3. Moreover, the catalase activity was found to be responsible for ROS scavenging in *MdSUMO2* RNAi plants. In addition, the accumulated protein of MdDREB2A caused up-regulated heat-responsive genes contributed to the thermotolerance of *MdSUMO2* RNAi plant, too. These findings will provide the candidate genes for future apple breeding in confronting the extreme climate change.

## Materials and methods

### Plant material and growth conditions

The experimental material used for gene cloning was ‘Golden Delicious’ (*Malus* × *domestica* Borkh. cv. Golden Delicious), which grown in Northwest A&F University, Yang ling, Shaanxi (34°20′N, 108°24′E). Leaves of ‘Golden Delicious’ was used for RNA extraction, RT-PCR, and gene cloning. For apple transformation, the wild type GL-3, a progeny of 'Royal Gala' (*Malus* × *domestica*), was subcultured on Murashige and Skoog (MS) medium (4.43 g/L MS salts, 30 g/L sucrose, 0.2 mg/L 6-BA, 0.2 mg/L IAA and 7.5 g/L agar, pH 5.8) under long-day condition (14 h light [cool white, ~ 100 umolm^−2^ s^−1^, T5 LED batten]: 10 h dark) for 4 weeks at 25 °C (Xie et al. 2018). *MdSUMO2* RNAi plants were produced as described in a previous study (Li et al., 2022a). The transgenic plants and GL-3 were rooted, transplanted into substrate (Pindstrup, Denmark), and grown in a growth chamber at Northwest A&F University (16 h light: 8 h dark, 25 °C, ~ 55% relative humanity).

### RNA extraction and qRT-PCR analysis

RNA extraction and quantitative reverse transcription PCR (qRT-PCR) analysis were performed as described previously (Xie et al. 2018).

The extracted RNA was detected by agarose gel electrophoresis and the concentration was determined by UV spectrophotometer (Thermo Nanodrop 2000). For qRT-PCR analysis, approximately 1 ug of RNA was reverse transcribed with oligo-dT to first-strand cDNA using a RevertAid First Strand cDNA Synthesis Kit (Thermo Scientific, K1622, USA). qRT-PCR was performed on the CFX96 real-time system (Bio-Rad, USA), using a ChamQ Universal SYBR qPCR Master Mix (Vazyme, C601, China). Primers used in this study are list in Table 1.

**Table 1:** Sequences of primers used in this study

Gene Name	Forward Primer	Reverse Primer	Purpose
*MdSUMO2C*	GCACAGAAGAAACCCCTGGAT	CACTTCATTACCATCCTGCCCT	qRT-PCR analysis
*MdSUMO2A*	GTGTTTGGAAGTGGTGGTAGCTC	TCTTGTCCTCCTCCTGGTTCG	qRT-PCR analysis
*MdSUMO2B*	CGACTCCGCAGCAAGAAGAG	CTTCAGCTGAGTGCTTCGC	qRT-PCR analysis
*MdSUMO2-RNAi*	GGGGACAAGTTTGTACAAAAAAGCAGGCTTCTCAGGCGTCACGAACCAGG	GGGGACCACTTTGTACAAGAAAGCTGGGTCGCTGGGTACTTCTCTTGATCCTGA	Plasmid construction of *MdSUMO2* RNAi
*MdHSFA3*	CGGTCAACTATGATGTGCCA	GGCCACTTATCAATGCCTGA	qRT-PCR analysis
*MdHSP18.2*	TTCGAATTCACACCCAGCAA	GATGTCCAGAGAGAAGGGGT	qRT-PCR analysis
*MdHSP26.5*	CAGGCTGCTTGACAACCTAA	CCAGAAACCCGTCATGAACA	qRT-PCR analysis
*MdHSP70*	CAAGATGTACCAGGGTGGTG	AAATACACATTCACTCGCTTCA	qRT-PCR analysis
*MdCYP18-1*	TACAAGGTGGAGACCCAACG	TAGATGTGCCGCCTTTGCAT	qRT-PCR analysis
*MdTLP1*	GACACATGCAAGCCGAGTTC	TCGACGACTTCACACTGGTC	qRT-PCR analysis

### Thermotolerance assays

Thermotolerance stress was performed as described previously (Guan. et al. 2013). For analyzing the expression patterns of *MdSUMO2s* under heat stress, one-month-old GL-3 plants were put into a growth chamber with 45 °C for 120 min. For analyzing the phenotype of one-month-old apple plants, GL-3 and *MdSUMO2* RNAi transgenic apple plants with a consistent growth state were exposed to 45 °C for 8 h and allowed to grow for an additional 14 d. The leaves collected at 120 min were used for gene expression and protein analysis. The 3-month-old GL-3 and *MdSUMO2* RNAi transgenic apple plants were exposed to 40 °C for 7 d in outside environment. In the summer, the 8-month-old GL-3 and *MdSUMO2* RNAi transgenic apple plants grew in greenhouse suffered from high temperature at noon when the temperature reached to 50 °C, and the treatment lasted from 11:00 to13:00 for one month.

### Determination of H_2_O_2_ content, CAT activity, MDA content and ion leakage

H_2_O_2_ content was performed using a Hydrogen Peroxide Assay Kit (Comin, H2O2-1-Y, China).

CAT activity and MDA content were measured as described previously (Niu et al. 2022). For CAT activity and MDA content measurement, approximately 0.1 g leaf was used to extract. The extraction buffer includes 100 mM phosphate buffer [pH = 7.0], 1 mM ethylenediaminetetraacetic acid [EDTA], 0.1% Triton-X-100, and 1% polyvinyl pyrrolidone [PVP].

The electrolytic leakage assay was performed as previously described (Niu et al. 2022). Briefly, the leaves were collected from one-month-old GL-3 and *MdSUMO2* RNAi transgenic apple plants. The leaves were cut into leaf discs and placed in a glass tube. Then the discs were soaked in deionized water for 12 h and the tubes were then placed into the high-temperature aqua bath cycle instrument (Thermo Fisher PC200-A40) with the following heat temperature regime: successive 4 °C increases each hour from 23 °C to 35 °C, then hold at 35 °C for 1 h; then successive 4 °C increases at 1-h intervals, then hold at 39 °C for 1 h; then successive 4 °C increases at 1-h intervals, then hold at 43 °C for 1 h. Finally, the discs in tubes were boiled at 100 °C for 30 min and the conductivity was determined using a benchtop conductivity meter (Thermo Scientific, STARA2120, USA). Experiments were performed at least three times.

### In vivo SUMOylation and ubiquitination analysis

The SUMOylation and ubiquitination analysis were performed as previously described (Li et al., 2022b). In brief, the samples of analysis were collected at 0 h or 0.5 h or 1 h or 2 h under heat stress. Then the total proteins of the samples were extracted by protein extraction buffer (50 mM Tris–HCl, 150 mM NaCl, 2 mM EDTA, 1% Np-40, 10% Glycerin, 1 mM PMSF, 5 mM DTT, pH 7.5). Finally, the proteins were immunoprecipitated with anti-SUMO (ab5316, Abcam) or anti-Ubiquitin (P4D1, Cell Signaling Technology®) antibodies.

## Statistical analysis

For statistical analysis, the Student’s *t* test was used by using Prism 8.0 software (GraphPad Software, USA). Differences were identified significant if *P* < 0.05 or 0.01.

## Data Availability

All data generated or analyzed during this study are included in this published article.

## Abbreviations

CAT: Catalase
DAB: 3,3'-Diaminobenzidine tetrahydrochloride
DRE: Dehydration responsive element
DTT: Dithiothreitol
EDTA: Ethylene diamine tetraacetic acid
MDA: Malondialdehyde
NBT: Nitro-blue tetrazolium
PMSF: Phenylmethylsulfonyl fluoride
PVP: Polyvinyl pyrrolidone
qRT-PCR: Quantitative reverse transcription PCR
